# Severe Hypokalemic Paralysis Following Rituximab Infusion in a Patient With Microscopic Polyangitis

**DOI:** 10.7759/cureus.84750

**Published:** 2025-05-24

**Authors:** Yousuf Sherwani, Ayham Alsaab, Mohan Sengodan

**Affiliations:** 1 Internal Medicine, Medical City Arlington, Fort Worth, USA; 2 Internal Medicine, Medical City Arlington, Arlington, USA; 3 Internal Medicine, Medical City Fort Worth, Fort Worth, Texas, USA

**Keywords:** acute flaccid paralysis, hypokalemia, microscopic polyangeitis, physical therapy, prolonge qtc, rituximab therapy, sjogren's syndrome

## Abstract

Rituximab is an anti-CD20 monoclonal antibody that has become an increasingly popular choice for the treatment of various autoimmune diseases. Various adverse effects have been attributed to the use of rituximab. Hypokalemia is one rare adverse reaction that is under reported in the literature and is life threatening. Here we report the case of a patient with symptomatic hypokalemia after IV rituximab infusion. Acute hypokalemia should be taken into account by healthcare professionals as a possible side effect after rituximab infusion.

## Introduction

Rituximab is a human-mouse chimeric anti-CD20 monoclonal antibody used in the treatment of autoimmune disease and multiple hematological malignancies [1,2]. Rituximab targets CD20 on B cells and induces B-cell-depletion by triggering the immune system through complement-dependent cytotoxicity and antibody-dependent cell-mediated cytotoxicity [3]. Rituximab has been becoming an increasingly popular treatment option in recent years. Multiple side effects of rituximab have been cited in the literature including infusion reactions, increased risk of infection, cytopenia, tumor lysis syndrome, cardiac arrythmia, nausea, vomiting, diarrhea, headache, mood disorder, fever, myalgia, fatigue, and drug-drug interaction [4]. However, a rarely reported side effect of rituximab infusion is acute hypokalemia with clinical manifestations. 

It is imperative to recognize hypokalemia as complications can be fatal. These complications include but are not limited to cardiac arrythmia, cardiac failure, muscle weakness, paralysis, intestinal paralysis, and respiratory compromise [5]. Hypokalemia causes weakness by disrupting electrical potential across the muscle membrane, which leads to impaired action potentials and impaired muscle excitability. Impaired action potentials lead to impaired nerve impulses disrupting muscle contractions that are necessary for muscle function [6]. Hypokalemia as a potential side effect of rituximab that has only been described a few times in the literature. One such report is a case report from 2020 that describes symptomatic hypokalemia in an 18-year-old female with steroid-dependent nephrotic syndrome after her sixth rituximab infusion, with K dropping from 4.0 mEq/L prior to infusion to 2.3 mEq/L at the time of symptoms [7]. Another case report in 2023 described hypokalemia after rituximab infusion for a patient with renal membranous nephropathy and nephrotic syndrome [8]. In both of these reports patients had underlying nephropathy and were given rituximab for treatment of renal pathology. However, we have yet to find a case report that describes hypokalemia after rituximab infusion for the treatment of autoimmune disease without nephrotic syndrome. Here we present a case of severe hypokalemia in a patient after receiving rituximab infusion for treatment of her underlying rheumatologic diseases.

## Case presentation

A 53-year-old female patient presented to the emergency department complaining of diffuse muscular weakness that rapidly progressed over the course of three to four hours following her fourth rituximab infusion that morning. Her past medical history included microscopic polyangitis, Sjogren's syndrome, type 1 renal tubular acidosis, and rheumatoid arthritis. The patient had previously been noted to have regular potassium levels from routine outpatient labs. Emergency medical service (EMS) was called after weakness progressed to the point that she could not stand from a seated position and had to be "carried to the hospital". She denied any sensory deficits, numbness, or paresthesia. There was no recent history of nausea, vomiting, or diarrhea. The patient previously presented to our rheumatology outpatient clinic for worsening peripheral neuropathy, lower extremity edema ,and erythematous rash present on her lower extremities. The patient was referred to dermatology for biopsy of the rash. Biopsy results revealed leukoctyoclastic anti-neutrophil cytoplasmic antibody (ANCA) vasculitis. The patient was subsequently given a steroid taper and started on rituximab, with 1 g infusion once per week for four weeks. On this regiment the patient’s lower extremity edema, peripheral neuropathy, and erythamatous rash improved. The patient tolerated four weeks of treatment with this regiment.

**Figure 1:**
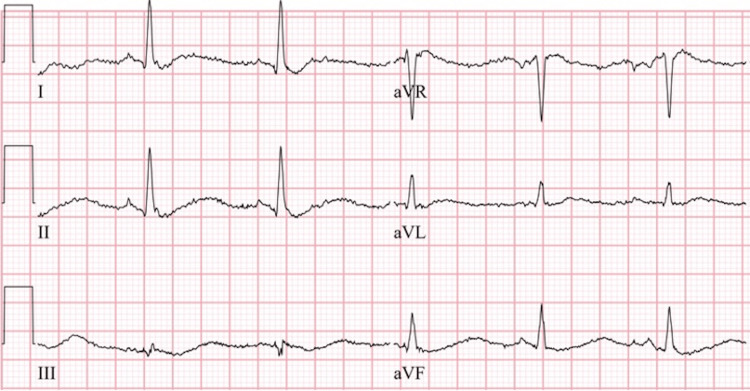
EKG showed normal sinus rhythm with sinus arrhythmia, a rate of 73, diffuse repolarization abnormalities, flattened T waves, and a prolonged QT interval in the limb leads EKG: Electrocardiogram

**Figure 2:**
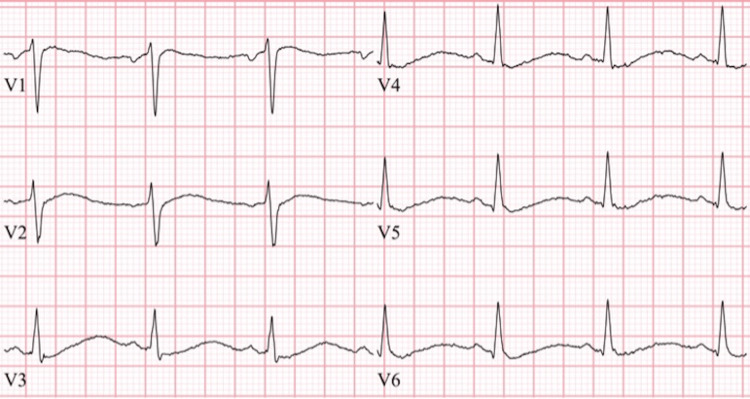
EKG showed normal sinus rhythm with sinus arrhythmia, a rate of 73, diffuse repolarization abnormalities, flattened T waves, and a prolonged QT interval in precordial leads EKG: Electrocardiogram

On arrival at the emergency room, vitals were: heart rate 86 beats per minute, respiratory rate 16 per minute, blood pressure 119/75 mmHg, and oxygen saturation 99% on room air. Electrocardiogram (EKG) showed changes consistent with hypokalemia (Figures 1, 2). On physical exam, cranial nerves were intact but the patient had slowed reflexes and 2/5 muscle strength in both upper and lower extremities with intact muscle tone. Initial blood work was significant for hypokalemia at a level of 1.3 mmol/L, hypophosphatemia to 0.3 mg/dL, and hyperchloremic, non-anion gap metabolic acidosis with bicarbonate of 14 mmol/L and chloride of 119 mmol/L. Magnesium level was slightly elevated at 2.5 mg/dL. Urine anion gap was positive (+11) and urine pH 7.0 despite systemic acidosis. Workup of hypokalemia was complicated by urine studies showing spot urine K of 8 meq/L (Table 1).

**Table 1:** Admission blood work results and reference ranges

	Test Results	Reference Range
Sodium	136 meq/L	135-145 meq/L
Potassium	1.3 meq/L	3.5-5.0 meq/L
Bicarbonate	14 meq/L	22-26 meq/L
Magnesium	2.5 mg/dL	1.7-2.2 mg/dL
Chloride	119 meq/L	96-106 meq/L
Phosphate	0.3 mg/dL	2.5-4.5 mg/dL
Urine pH	7.0	4.5-8.0
Urine anion gap	11 meq/L	-10 to 10 meq/L
Urine spot potassium	8 meq/L	15-20 meq/L

She was admitted to the ICU for hypokalemia and aggressive IV electrolyte repletion. The patient received a total of 535 meq of potassium via oral and IV routes over three days with progressive and rapid improvement in muscle weakness. As the patient’s potassium normalized, her weakness improved accordingly until all symptoms were gone (Table 2). The cause of presentation was determined to be likely due to rituximab infusion. 

**Table 2:** Potassium level and associated symptoms on admission to twelve days out since admission

Days since Presentation	Potassium level (meq/L)	Symptoms
0	1.8	Profound diffuse weakness
2	3.2	Mild lower extremity weakness
3	4.0	Complete resolution of symptoms
12	4.1	Remains asymptomatic

## Discussion

Rituximab has been an emerging treatment for multiple autoimmune diseases, lymphomas, and nephrotic syndrome. It has allowed for far less steroid dependency for patients suffering from refractory autoimmune disease. The most common side effects of rituximab administration are acute infusion-related reactions consisting of fever, chills, rash, and pruritus [9]. Although rare, hypokalemia is a potentially life threatening adverse reaction to rituximab that can lead to cardiac arrhythmia and cardiopulmonary compromise. The exact mechanism of how rituximab leads to hypokalemia is unknown. Potential hypotheses do exist. It has been found that rituximab significantly decreases intracellular Ca2+ concentration and this deactivates Ca2+ gated potassium (IK) channels [10]. Additionally complement-dependent cytotoxicity which induces apoptosis of B lymphocyte by stimulating FcγRIIB receptors concurrently leads to the inhibition of potassium v1.3 channels [11]. There have also been reports of rituximab’s effect on potassium N member 4 (KCNN4) channels, though the effect of rituximab on hypokalemia is still being investigated [12]. Hypokalemia can have multiple clinical manifestations. In our patient paralysis was the main symptom. Hypokalemia induces weakness by disrupting electrical potential across the muscle membrane which leads to impaired action potentials and impaired muscle excitability. Impaired action potentials lead to impaired nerve impulses disrupting muscle contractions that are necessary for muscle function. Correction of the potassium levels leads to resolution of weakness [6]. 

We did rule out other causes of hypokalemia. Common causes of hypokalemia including vomiting and diarrhea were ruled out as the patient had no history of these symptoms. Medication review did not yield any other medication that could explain the profound hypokalemia. Other differentials included exacerbation of type 1 renal tubular acidosis, Gitelman syndrome, thyrotoxic periodic paralysis, and primary hyperaldosteronism. The patient's TSH and T4 levels were within normal limits and thus thyrotoxic periodic paralysis was unlikely to be the cause of our patient's symptoms. Furthermore, although Gitelman syndrome and primary hyperaldosteronism seemed like potential causes of hypokalemia in our patient, a low urine spot potassium would be an unlikely finding in either pathology. In the case of primary hyperaldosteronism, hypernatremia would also be expected which was not seen. Finally, a promising cause of hypokalemia in our patient was due to type 1 renal tubular acidosis. The acidosis seen in this patient, along with the low phosphate level, would be typical of renal acidosis. However, the low urine spot potassium does not suggest renal wasting of potassium. There may have been a component of exacerbation of renal tubular acidosis in our patient however we believe that the profound hypokalemia was caused by IV rituximab. The presence of paralysis shortly after rituximab infusion further validates this conclusion. The patient's potassium remained normal weeks later during outpatient follow up suggesting medication induced hypokalemia.

Hypokalemia can be abrupt in patients on rituximab therapy. Our patient had normal blood potassium and was completely asymptomatic in the days before her rituximab infusion. Patients should be warned about hypokalemia as a potential side effect. If symptoms of dizziness, fatigue or weakness are experienced by patients then prompt blood work should be obtained to quantify the patient's serum electrolytes. Hypokalemia caused by rituximab can be corrected effectively with both PO and IV supplementation. Symptoms are likely to resolve as potassium levels normalize. However if hypokalemia is not promptly treated then the consequences can be fatal. Our patient was showing signs of hypokalemia on EKG and was at increased risk of a variety of cardiac arrhythmias including ventricular tachycardia, ventricular fibrillation, atrial flutter, torsades de pointe, and many others [13]. Retrospective studies as well as clinical trials will need to be performed to better quantify the risk of hypokalemia with rituximab infusion. Early monitoring for signs and symptoms of rituximab-induced hypokalemia can reduce morbidity and mortality.

## Conclusions

In conclusion, as rituximab becomes an exceedingly popular treatment option for autoimmune disorder, rituximab-related adverse reaction will be more common as well. One under-reported side effect is hypokalemia. Early monitoring and effective management of hypokalemia are important for patients who receive rituximab-based therapy.
